# The Long-Term Impacts of an Integrated Care Programme on Hospital Utilisation among Older Adults in the South of England: A Synthetic Control Study

**DOI:** 10.5334/ijic.6475

**Published:** 2023-08-17

**Authors:** Paul Seamer, Therese Lloyd, Stefano Conti, Stephen O’Neill

**Affiliations:** 1 The Strategy Unit, NHS Midlands & Lancashire Commissioning Support Unit, Birmingham, UK; 2 Improvement Analytics Unit, The Health Foundation, London, UK; 3 Population Health Management, NHS England, London, UK; 4 Department of Health Services Research and Policy, London School of Hygiene & Tropical Medicine, London, UK

**Keywords:** integrated care, hospital utilisation, synthetic control, long-term, older people, health care, England

## Abstract

**Introduction:**

Reducing hospital use is often viewed as a possible positive consequence of introducing integrated care (IC). We investigated the impact of an IC programme in North East Hampshire and Farnham (NEHF), in southern England, on hospital utilisation among older adults over a 55 months period.

**Method:**

We used a Generalised Synthetic Control design to investigate the effect of implementing IC in NEHF between 2015 and 2020. For a range of hospital use outcomes, we estimated the trajectory that each would have followed in the absence of IC and compared it with the actual trajectory to estimate the potential impact of IC.

**Results:**

Three years into the programme, emergency admission rates started reducing in NEHF relative to its synthetic control, particularly those resulting in overnight hospital stays. By year 5 of the study overall emergency admission rates were 9.8% lower (95% confidence interval: –17.2% to –0.6%). We found no sustained difference in rates of emergency department (ED) visits, and average length of hospital stay was significantly higher from year 2.

**Conclusion:**

An IC programme in NEHF led to lower than estimated emergency admission rates; however, the interpretation of the impact of IC on admissions is complicated as lower rates did not appear until three years into the programme and the reliability of the synthetic control weakens over a long time horizon. There was no sustained change in ED visit rates.

## Introduction

Many global health systems are struggling to meet the needs of growing numbers of older people and people living with multiple long-term conditions. Integrated care (IC) is widely viewed as offering a potential solution to some of these challenges [1], and the integration of care, both within the healthcare sector and also between the health and social care sectors, has become a major focus of policy in many developed countries.

Reducing hospital use is often viewed as a possible positive consequence of introducing IC [2]. However, the overall message emerging from most impact evaluations is that the evidence on benefits is rather mixed [3]. There is some evidence of improved patient-perceived quality of care, increased patient satisfaction, and of improved access to care, but evidence for other outcomes, including system-wide impacts on primary care, secondary care, and health care costs is limited [4]. Furthermore, some studies have found people receiving IC services using some types of hospital care more than matched controls [5,6,7,8,9].

Several reasons have been put forward for the failure of evaluations of IC to produce the expected results of reduced hospital admissions. A common criticism is that programme evaluations have often been informed by unrealistically short follow-up times, and that given sufficient time IC programmes would eventually start to bend the demand curve for hospital care [10]. A large umbrella review of systematic reviews and meta-analyses identified that most studies lasted no more than 12 months [11].

In 2014 the health service in England set out an overall vision for reform, based around the creation of ‘new care models’ that would seek to break down barriers between care sectors, and provide IC to populations [12]. The focus of these plans was the establishment of ‘Vanguard’ sites to design and test locally driven prototypes for integrating health and social care services [13]. The overall scale of the Vanguard piloting programme was large: in total, 50 vanguard sites, covering a population of around five million were selected across England. Vanguard sites received additional funding to test out these new models of care and were backed by a national support programme. Initially, Vanguard sites were granted freedom to select their own outcomes, but there was an expectation from those leading the national programme that tackling fragmentation, duplication and poor co-ordination of care would reduce hospital utilisation.

Additional funding allowed health and care organisations in North East Hampshire and Farnham (NEHF) to implement a range of initiatives between 2015 and 2018 as part of their ‘Happy, Healthy, at Home’ Vanguard programme. The most significant of these was the development of five integrated care teams (ICTs) covering the whole area. The study start date, August 2015, was selected to coincide with the introduction of the ICTs.

The primary objective of the ICTs was to reach the most vulnerable patients including those with complex care needs or at greatest risk of an adverse event (eg unplanned admission to hospital or premature admission to a nursing home). Professionals from primary, community, mental health and social care services were tasked with working together to plan and deliver a coordinated care package for each patient referred to the team.

Table 1 summarises some of the other main changes to models of care that were implemented in NEHF. After the additional funding ended in March 2018, the ICTs and other major components of the programme continued to operate and were funded from local health budgets. The coronavirus pandemic and its dramatic effect on all types of hospital activity meant the study could not continue beyond February 2020.

**Table 1:** Timeline of major changes to models of care in NEHF.


**2015 April to 2018 March**	**Funding:** The NEHF Vanguard received £14.3 million of national non-recurring funding spread across three years (£3.4 m in 2015–16, £5.3 m in 2016–17, and £5.6 m in 2017–18). The primary organisation responsible for commissioning health services for the local population (NEHF clinical commissioning group) had an annual budget of £238 m for the financial year 2015–16.

**2015 July**	**Integrated care teams:** Five multi-disciplinary teams made up of professionals from primary care, community care, mental health, social care, and the voluntary sector working together to provide more coordinated care through a single care planning process.

**2015 August**	**Study starts**

**2016 November**	**Enhanced recovery at home service:** A service to facilitate timely discharge and a seamless transition to ongoing care following an unplanned admission to hospital, or to avoid hospital for those who can be supported to remain at home.

**2016 November**	**Ambulatory emergency care:** A new unit at the main local hospital providing rapid assessment, diagnosis, and treatment so that, if clinically safe to do so, patients presenting at hospital with relevant conditions can return home the same day their care is provided. Patients treated in the new unit were recorded as being admitted to hospital even if discharged later the same day.

**2017 February, and 2017 June**	**Primary care-led urgent care centres:** two new centres providing urgent, same day primary care advice and treatment to patients registered with local GP practices.

**2017 March**	**Rapid home response service:** A support service provided by specially trained community paramedics for patients at immediate risk of hospital admission. The team also provides bridging support to enable patients to stay at home while care packages are arranged.

**2017 April**	**111 GP triage service:** A service aimed at reducing the number of non-urgent referrals to ED from the national 111 non-emergency medical helpline by offering patients a call with a GP within 15 minutes of their 111 call. NHS 111 is a national service, but the service model associated with this initiative was different to that being operated in most other parts of England.

**2020 February**	**Study ends**


NEHF is situated in the South of England and is home to a population of 225,000 people. The planning and funding of health services for the NEHF population is overseen by a single clinically led organisation, which includes all the general practices in the area. Compared with the national average, people living in NEHF enjoy higher life expectancy and lower levels of socioeconomic deprivation.

The aim of this study was to investigate the effect of implementing an IC programme in a single health economy in the South of England on the rate of emergency hospital use among its older adult population. Specifically, we wished to explore the hypothesis that IC initiatives require more time to reach maturity than is typically allowed for in impact evaluations, and that over a longer timeframe reductions in population hospital use would follow. Maximising the learning from the Vanguard programme has taken on greater significance in light of ambitious plans to deliver more integrated care within the new Integrated Care Systems in England, set to be implemented nationally from April 2022.

## Methods

### Approach

We used a Generalised Synthetic Control (GSC) design [14], an approach especially well-suited to population-level studies when random assignment to an intervention (in our case the IC Vanguard programme) is impractical, and a data driven approach is needed to construct appropriate comparison cases [15]. For each outcome variable, we estimated the trajectory it would have followed in the absence of IC and compared it with the actual trajectory to estimate the potential impact of IC. The counterfactual, or synthetic control, was constructed based on a weighted combination of general practices, drawn from elsewhere in England, that were not exposed to the Vanguard programme. If the synthetic control closely tracks the actual trajectory during the pre-intervention period, then we can have confidence in the validity of the estimated counterfactual trajectory during the post-intervention period. A key benefit of this approach is that it removes the need to identify individual comparison general practices that are sufficiently similar to practices in NEHF [16].

Our study period was 79 months from the year 2014 to the year 2020, comprising of 24 months before (July 2013 to July 2015) and 55 months following (to February 2020) the start of the IC programme. The long follow-up time, of more than 4.5 years, meant we could see whether the effect of IC changed over time and test the hypothesis that it may take several years for IC initiatives of this sort to bring about reductions in hospital utilisation.

At the time the IC programme was introduced in NEHF in August 2015 the local population was served by 24 general practices. We excluded from the analysis three practices that closed during the follow-up period and a further two practices with incomplete hospital activity data leaving 19 intervention practices (see appendix for further details).

### Data

Hospital activity data were obtained from the Secondary Uses Service, a national, person-level database containing pseudonymised details of all admissions, ED visits and outpatient appointments at NHS hospitals in England. Data were aggregated across patients by general practice and structured as monthly series for every practice retained in the study.

Sociodemographic background characteristics (eg population size, age profile, deprivation levels, prevalence of long-term conditions) were collected from publicly available sources and structured as monthly series for every general practice in England.

### Outcomes

To obtain a complete picture of the effect of IC on emergency hospital use we investigated six outcome variables.

- Emergency department (ED) visits to type-1 departments — major units, providing a consultant-led 24-hour service with full resuscitation facilities (monthly rate)
- All-cause emergency admissions (monthly rate)
- Average length-of-stay for overnight emergency admissions (days)

*Subsets of all-cause emergency admissions*

- Overnight all-cause emergency admissions (monthly rate)
- Emergency admissions for chronic ambulatory care sensitive conditions (CACSC) (monthly rate)
- Emergency admissions for urgent care sensitive conditions (UCSC) (monthly rate)

CACSC refers to hospitalizations for chronic conditions that would have been possible to avoid by timely and effective use of primary or community care. UCSC refers to acute conditions that should not usually require hospital admission. In this study both sets of conditions were coded using the categorisation used by the English National Health Service (NHS) [17].

Data for event outcomes were collected at patient level before aggregating to monthly counts at general practice level and used in the model as monthly rates per 10,000 registered patients. Average length of stay was calculated as the sum of overnight bed-days for emergency admissions occurring in each month over the count of those admissions.

All outcome variables were analysed separately for patients of age 65 years or over and patients of age 18 years or over. Our primary focus was hospital use among older adults as this group best reflected the age profile of patients targeted by the ICTs, the highest-profile initiative in the IC programme [8]. However, because adults younger than 65 years of age could be referred to the ICTs, and to understand the overall effect of all the Vanguard initiatives and wider changes to the health and care system in NEHF, we also examined hospital use among the whole adult population.

### Selecting the control group

Across England there are more than 7,000 general practices. To narrow down the pool of potential control practices we identified and excluded practices from areas that were most dissimilar to NEHF or in areas that were also participating in the Vanguard programme. From the remaining pool of c.1,000 general practices, we selected the 200 general practices that were most similar to the NEHF practices at baseline, across a range of sociodemographic variables and measures of hospital utilisation in the 24-months leading up to the start of the IC programme, to form the control group for our main analysis. More information on how the control group was selected and a complete list of the variables used is included in the appendix.

### Statistical analyses

We used a version of the Synthetic Control method that has been shown to perform better than alternatives across a range of challenging settings typically faced in health economic and policy evaluation [18]. The GSC method constructs the counterfactual in three steps. Firstly, a fixed number of latent factors are estimated using the control group (those practices that did not take part in the Vanguard programme). Secondly, those factors are weighted to model the path of each outcome variable during the pre-intervention period (the period before IC was introduced). Thirdly, that model is used to estimate the counterfactual path of the outcome variable during the post-intervention period. Confidence intervals are estimated using a parametric bootstrap procedure based on how reliably the model predicts the path of each control practice when it’s assumed to be an intervention practice. The analysis was carried out using the R platform (version 4.0.3), with the functions provided by the gsynth package (version 1.1.7) [19,14].

We applied regression-based risk-adjustment to account for time-varying differences in the characteristics of general practice populations and of patients admitted to hospital. More information on the risk adjustment and a complete list of the variables used is included in the appendix. Under reasonable modelling assumptions, the GSC approach reports efficient estimates with low bias even in the presence of time-varying unobserved confounders and heterogenous (ie sub-group level) intervention effects [18].

We report estimates of the impact of IC for each financial year (or part thereof) of the post-intervention period by averaging across the monthly effect estimates.

## Results

**Figure 1:**
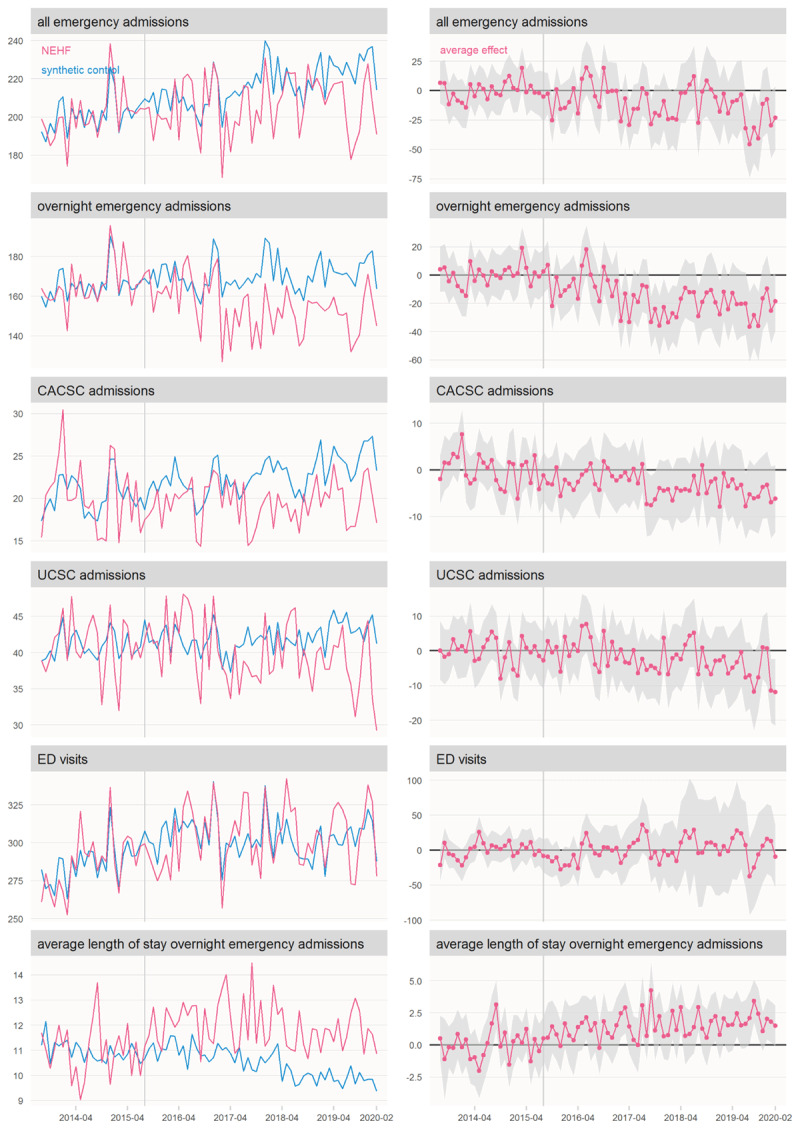
Counterfactual and intervention averages, and average effect, 65+ year-old population (admissions outcomes and ED visits are rates per 10,000 population per month; average length of stay is days). CACSC = chronic ambulatory care sensitive conditions, UCSC = urgent care sensitive conditions.

For each outcome variable Figure 1 shows the average intervention and counterfactual trajectories, and the average monthly intervention effect. The counterfactual trajectories track the intervention trajectories during the pre-intervention period reasonably closely, suggesting that the adopted models are able to predict reliable counterfactuals for the post-intervention period. The effect plots represent the gap between the intervention and counterfactual trajectories and include 95% confidence intervals.

**Figure 2:**
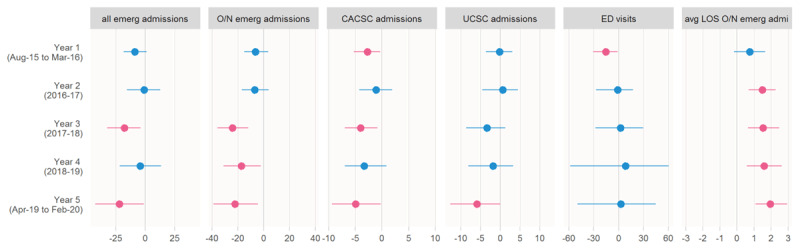
Average effect (difference between outcome observed in NEHF and estimated counterfactual), 65+ year-old population. Admissions outcomes and ED visits are rates per 10,000 population per month; average length of stay is days. Red = confidence interval does not contain zero, blue = confidence interval contains zero. CACSC = chronic ambulatory care sensitive conditions, UCSC = urgent care sensitive conditions.

Table 2 and Figure 2 show yearly intervention effect estimates. These reveal a consistent pattern in the average effect across the four admissions-related outcomes. For all emergency admissions, overnight emergency admissions and the two sub-groups of potentially avoidable admissions (CACSC and UCSC) the average gap between actual and counterfactual rates changed relatively little in the first two years after the introduction of IC, but from year three onward the gap showed signs of widening. In particular, overnight emergency admissions were significantly lower in NEHF than in the synthetic control in years three, four and five. By year five all four admissions outcomes were significantly lower in NEHF than in the counterfactual: overall emergency admission rates were 9.8% lower (95% confidence interval: –17.2% to –0.6%), overnight emergency admissions were 12.9% lower (95% confidence interval: –20.5% to –2.9%), CACSC were 19.9% lower (95% confidence interval: –32.0% to –1.2%), and UCSC were 13.5% lower (95% confidence interval: –25.2% to 0.0%).

**Table 2:** Average effect (difference between NEHF and estimated counterfactual), 65+ year-old population. Admissions outcomes and ED visits are rates per 10,000 population per month; average length of stay is days. CACSC = chronic ambulatory care sensitive conditions, UCSC = urgent care sensitive conditions.


OUTCOME VARIABLE	YEAR 1 AUG–15 TO MAR–16	YEAR 2 2016–17	YEAR 3 2017–18	YEAR 4 2018–19	YEAR 5 APR–19 TO FEB–20

All emergency admissions, rate					

*Difference*	–8.9 (–18.3 to 1.11)	–1.0 (–15.5 to 12.8)	–17.7 (–32.3 to –4.1)	–4.4 (–21.7 to 13.3)	–22.1 (–42.4 to –1.2)

*Rel. difference (%)*	–4.2 (–8.3 to 0.6)	–0.5 (–7.0 to 6.6)	–8.1 (–13.7 to –2.0)	–2.0 (–9.2 to 6.6)	–9.8 (–17.2 to –0.6)

Overnight emergency admissions, rate					

*Differenc*e	–6.3 (–14.8 to 3.7)	–6.9 (–17.0 to 4.1)	–24.1 (–35.7 to –11.9)	–17.1 (–31.1 to –2.1)	–22.3 (–38.8 to –4.5)

*Rel. difference (%)*	–3.7 (–8.3 to 2.3)	–4.1 (–9.6 to 2.6)	–14.0 (–19.4 to –7.4)	–10.1 (–17.0 to –1 .4)	–12.9 (–20.5 to –2.9)

CACSC admissions, rate					

*Difference*	–2.7 (–5.3 to –0.3)	–1.1 (–4.3 to 1.9)	–4.0 (–6.9 to –0.8)	–3.4 (–6.9 to 0.8)	–5.0 (–9.4 to –0.2)

*Rel. difference (%)*	–12.6 (–21.8 to –1.6)	–5.2 (–17.5 to 10.5)	–17.8 (–27.2 to –4.2)	–14.8 (–26.5 to 4.4)	–19.9 (–32.0 to –1.2)

UCSC admissions, rate					

*Difference*	–0.2 (–3.7 to 3.1)	0.6 (–4.6 to 4.5)	–3.3 (–8.7 to 1.3)	–1.8 (–8.1 to 3.3)	–5.9 (–12.7 to 0.0)

*Rel. difference (%)*	–0.4 (–8.0 to 8.0)	1.4 (–9.9 to 11.9)	–8.1 (–18.6 to 3.6)	–4.4 (–16.9 to 8.9)	–13.5 (–25.2 to 0.0)

ED visits, rate					

*Difference*	–15.4 (–30.8 to –1.3)	–1.3 (–27.3 to 17.6)	2.4 (–28.1 to 29.7)	8.4 (–58.2 to 60.4)	2.9 (–49.5 to 44.7)

*Rel. difference (%)*	–5.1 (–9.6 to –0.5)	–0.4 (–8.2 to 6.1)	0.8 (–8.4 to 10.7)	2.8 (–16.0 to 24.7)	0.9 (–13.8 to 16.9)

Average length of stay overnight emergency admissions, days					

*Difference*	0.7 (–0.2 to 1.7)	1.5 (0.7 to 2.3)	1.5 (0.7 to 2.5)	1.6 (0.6 to 2.6)	2.0 (1.1 to 2.9)

*Rel. difference (%)*	6.7 (–1.5 to 16.3)	13.7 (5.9 to 22.2)	14.4 (5.7 to 25.5)	16.0 (5.3 to 29.6)	19.9 (10.2 to 33.3)


ED visits among older adults in NEHF continued to follow a very similar trajectory to that of the counterfactual throughout the study period, suggesting IC had little or no effect on this type of activity.

From year two onward, average length of stay for overnight emergency admissions was significantly higher in NEHF than what we estimate it would have been in the absence of IC. By the final year of the study the difference was 2.0 days (95% confidence interval: 1.1 to 2.9).

As a secondary analysis, we examined hospital use among the population of age 18 years and over. In general, the effect estimates for this population followed a similar pattern to those for the older adult cohort. By year five overall emergency admission rates for this group were 11.7% lower (95% confidence interval: –18.4% to –2.4%) in NEHF than in the counterfactual. These additional results are presented in the appendix.

### Sensitivity analyses

When designing the study we made certain choices, for example regarding the length of the pre-intervention period, which variables to include in the risk adjustment, and the number of general practices to use in the control group. We performed sensitivity analyses to explore if our findings would have changed had we made different choices.

Sensitivity analyses confirmed that our findings were robust to changes in the duration of the pre-intervention period, the composition and number of units used to create the synthetic controls, and the use of risk adjustment.

## Discussion

In this study we have examined the impact on emergency hospital use among older adults following the introduction of IC in NEHF. A key aim for the programme was that a range of initiatives sharing the common objective of delivering more IC would bring about a reduced reliance at the population level on emergency care. The nature of the initiatives and their implementation meant we did not necessarily expect to see immediate effects.

Three years after the start of the programme we began seeing lower than expected emergency admission rates in NEHF. This was especially true for overnight emergency admissions which were significantly lower from year 3 onward. In year 5, for example, NEHF patients had on average 22.3 (95% confidence interval: –38.8 to –4.5) fewer overnight emergency admissions per 10,000 people per month, equivalent to a relative difference of –12.9% (95% confidence interval: –20.5% to –2.9%). Rates of admission were also significantly lower in year 5 for the CACSC and UCSC admission sub-groups. Average length of stay for overnight emergency admissions was, on average, significantly higher than the counterfactual estimate from year two onward. By the final year of the study the difference was 2.0 days (95% confidence interval: 1.1 to 2.9). Finally, ED visit rates continued to follow a similar path to the synthetic control, suggesting IC had little or no effect on this type of activity.

The pattern across most of our outcomes was that the average gap between NEHF and its synthetic control changed relatively little in the first two years, but from year three onward showed signs of widening. Eleven of fourteen significant results were in year three or later (Figure 2). While GSC is considered a strong method for health policy evaluation, over a long time horizon there is a greater risk of the modelled relationship between NEHF and the synthetic control breaking down. This means, that other things being equal, we must be more cautious in attributing differences between NEHF and its synthetic control to the impact of IC in later years than earlier in the study.

This study was not designed to estimate the relative contribution of the different initiatives introduced in NEHF as part of the Vanguard programme. From discussions with those involved in the programme the introduction of ICTs was considered to be the most significant change, but we can’t rule out the possibility that lower admission rates in later years were more a short-term response to later initiatives than a long-term effect of changes introduced at the start of the study.

We found no lasting difference in ED visit rates between NEHF and the counterfactual estimate. This is somewhat at odds with our finding of lower than estimated rates of emergency admissions from year 3. Typically, patients presenting at ED account for around 75% of all emergency admissions, with most of the remainder admitted directly following an urgent request from a GP [20]. Our analysis did not look at the proportion of ED visits that resulted in an admission, so we cannot determine if this changed differently over time in NEHF compared with the counterfactual.

If the proportion of ED visits ending in admission did evolve differently in NEHF compared with other areas in England, possible explanations include a change in the case-mix of patients presenting in ED or a change in the threshold for admission from ED. If the latter, this could have resulted from the interplay of several factors, including more confidence in community-based services to manage patients out of hospital or better access to rapid testing or specialist input within the hospital. The development of ambulatory or same-day emergency care was noted as a possible contributory factor behind a decrease in the case-mix adjusted odds of hospital admission via ED in England between 2010 and 2015 [21]. However, we do not think this was a factor in NEHF as, unlike in some areas, treatment in the Ambulatory Emergency Care unit at the main hospital serving NEHF was recorded as an emergency admission. The unit may, however, have contributed to lower overnight emergency admissions.

We found that the reduction seen in emergency admissions in NEHF was most noticeable for overnight admissions. At the same time, the average length of stay for overnight emergency admissions increased in NEHF whereas we estimated it would have decreased in the absence of IC. It may be that the reduction observed in overnight emergency admission rates was achieved by primarily avoiding overnight admissions for less serious conditions that would have otherwise resulted in only short lengths of stay. If true, this would have the effect of increasing the average length of stay among the remaining cohort of patients.

Because our primary analysis of the older persons cohort is nested within the secondary analysis of the all adult population at least some of the effect observed in the all adult group is driven by what happened in the older cohort. Looking at the all emergency admissions outcome, for both age groups the biggest effects occurred in year 3 and year 5. In both cases the difference in admission rates was greater for the older group, however, it was not so large that it completely dominated the effect in the all adult group. The total adult population in NEHF is about 4.3 times greater than the 65 and overs population meaning a fall in the older persons rate by 4.3 implies a fall of one in the all adult rate. These calculations suggest that the programme in in NEHF may have also contributed to reduced admissions among the under 65 population.

Long-term studies of the effect of IC are rare. However, one such study of an IC transformation programme in Mid-Nottinghamshire in England showed a delayed effect on hospital use with reductions in ED visits and emergency admissions compared to a control, but not until 5–6 years after the programme started and not within the subgroup of older people (those aged 65 years or over) [22]. A similar study investigating the effect of the Vanguard programme across two areas in the North West of England reported only tentative signs of an effect on hospital use across a 4.5-year follow-up [23].

Other studies have also looked at the effect of the Vanguard programme in England. A recent study looked at the effect of IC models on hospital use across 23 Vanguard sites, including NEHF. The authors reported a statistically significant reduction in emergency admission rates in the third year after implementation but found no change in total hospital bed-days [24]. Another study looked at IC models in two Vanguard sites, over a three-year follow-up, and found total costs of secondary care increased in both sites, but no effects on avoidable admissions, health status, or patient experience of care [25].

### Strengths and limitations

Robust long-term evaluations of IC programmes are rare [3]. A strength of this study is the extended follow-up period allowing the possibility to monitor changes in outcomes over a longer than usual timeframe. On the other hand, a longer follow-up increases the risk that the modelled relationship between NEHF and the synthetic control breaks down or that idiosyncratic shocks to the healthcare system impact differently on practices in the intervention and control areas. Our control practices were drawn from all over England, limiting the impact that a shock specific to any one area could have on our findings but the possibility cannot be completely ruled out. Nevertheless, sensitivity analyses indicated that our findings were robust to changes in the composition and number of practices used to create the synthetic controls.

The method we used in this study estimated the effect of the IC initiatives introduced in NEHF by creating a synthetic version of each general practice based on a weighted combination of similar practices drawn from elsewhere in England that were not part of the Vanguard programme. This method has been shown to perform better than commonly used alternatives in the health policy evaluation setting [18].

Unlike the original Synthetic Control method, GSC allows for the inclusion of time-varying observed covariates. We adjusted for known differences in the characteristics of general practice populations and of patients admitted to hospital. Under reasonable modelling assumptions, the GSC approach reports efficient estimates with low bias even in the presence of time-varying unobserved confounders and in situations where policy effects are modified by unobserved covariates [18].

The number of time-varying coefficients (also referred to as latent factors) is automatically selected by a built-in cross-validation scheme, reducing the risk of overfitting. However, imprecisely estimated factors and factor loadings (unit-specific intercepts) can lead to invalid results. For the outcome models when latent factors were identified we plotted estimated factor loadings (practice-specific intercepts) of both treated and control practices and checked the overlap. The estimated factor loadings of the treated practices were within the convex hull of the control practices, which indicates that the treated counterfactuals were produced by more reliable interpolations instead of extrapolations.

Confidence in causal inference comes from the ability of the modelling process to reliably estimate the path of the intervention practices during the pre-intervention period. The GSC method assumes that closeness of fit in the pre-intervention period is an indicator of the model’s ability to generate a reliable counterfactual in the post-intervention period. We note that the ‘fit’ in the pre-intervention period is better for some outcome variables than for others, but all outcomes passed tests for a placebo effect (ie a null effect during the pre-intervention period).

We used data sourced from a national, individual-level database and constructed our outcome measures to fit, as far as possible, with types of hospital activity where we were confident in the consistency of recording. However, we know that some types of activity are not always consistently recorded, for example same day emergency care, where the patient is treated and discharged within a few hours, is not always recorded as an admission to hospital [10].

We compared practices in NEHF with control practices drawn from all over England. It is highly likely that many of these practices were in areas where efforts were being made to integrate care independently of the Vanguard programme. However, these efforts are, to a greater or lesser degree, happening across the whole country, making it impossible to identify comparison practices in areas without any IC initiatives. We considered such areas to represent ‘standard care’, while lacking Vanguard funding and delivering a lower intensity of IC.

The scope of this evaluation is limited to the effect of the initiatives in NEHF on hospital resource use. We are unable to say anything about possible effects on other important aspects of patient care (eg patient or staff satisfaction, patient-reported outcomes or costs).

The coronavirus pandemic and its distorting effect on all types of hospital activity meant it was necessary to cut short the planned follow-up period and end the study in February 2020, rather than the planned study end date of July 2020.

This study was based in a single health economy that introduced a specific set of initiatives over several years; as such, the findings are not readily generalisable to other areas.

## Conclusion

Reducing demand for hospital services has often been one of the intended aims of IC programmes [26]. This study found that an IC programme in NEHF may have led to lower than estimated rates of emergency admissions. However, the interpretation of the impact of IC on admissions is complicated as lower rates did not appear until three years into the programme and the reliability of the synthetic control weakens over a long time horizon. We found no sustained change in ED visit rates, but from year two onward average length of stay for overnight emergency admissions was significantly higher than the counterfactual estimate.

Our findings and those from other studies looking at the impact of integrated care programmes suggest policy makers should not expect such initiatives to lead to short-term reductions in hospital use. Over a longer period of three to six years, our findings offer some encouragement that, given sufficient time, IC may moderate upward pressure on admissions. Future efforts should focus on measuring a wider range of costs and outcomes to generate the evidence needed on the cost-effectiveness of IC to support policy decisions.

## Additional File

The additional file for this article can be found as follows:

10.5334/ijic.6475.s1 Appendix. Supplementary materials.
