# Supplementary material for: The Long-Term Impacts of an Integrated Care Programme on Hospital Utilisation among Older Adults in the South of England: A Synthetic Control Study
Source: Int J Integr Care. 2023 Aug 17;23(3):10. doi: 10.5334/ijic.6475 (PMC10437138; doi:10.5334/ijic.6475)

APPENDIX

A. Additional Methods

Selecting the control group

A two-step process was followed to select practices for the control group.

1. Practices from regions that were most dissimilar to NEHF or in areas that were also participating in the Vanguard programme were identified and excluded.

2. From the remaining pool, the 10 most similar GP practices to each treated practice were selected to form the donor control group for the GSC analysis.

The variables used to assess similarity across regions in step one are listed in Supplementary Table 1. Since the variables are measured on different scales, they were first standardised using inter-decile range standardisation. Similarity was then measured by computing squared Euclidean distances between each region. Practices located within the most similar regions were selected until a pool of around 1,000 practices was reached.

Before proceeding with step two the following exclusions were applied:

- practices that opened or closed during the study period

- practices with registered patient population sizes outside the range of registered sizes in the treated group

- practices with patterns of key outcome variables indicative of reporting errors

- practices without records in both ED and inpatient datasets, so the same set of practices could be used across different outcomes

A similar process to that used in step one was then applied to identify the most similar practices to each treated practice. The variables used in step two are listed in Supplementary Table 2. Before assessing similarity, variables were weighted according to how predictive they are of the rate of emergency hospital admissions in the total population in the final year of the pre-intervention period, after adjusting for the other variables in the year prior to that. The weight given to each variable was determined by the absolute value of the corresponding T-test statistic, estimated from a regression of the rate of emergency hospital admissions in the year prior to the start of the intervention on the variables for the preceding year.

Similarity was measured by calculating the standardised mean difference (SMD) between all pairs of treated and control practices. SMD values were calculated using annual estimates (of the variables listed in Supplementary Table 2) for each of the two years prior to the start date. After ordering control units by decreasing overall similarity the following steps were applied:

- select the set of control practices that are nearest to each of the treated practices
- arbitrarily order these, excluding any duplicates
- add the control practices that are second nearest to each of the treated practices, excluding any practices already selected
- repeat, until all control units have been selected

This left a final list of control practices ordered by decreasing similarity with the treated practices. The top 100 'most similar' practices from this list were used in the GSC analysis.

Excluded practices

At the time the IC programme was introduced in NEHF in August 2015 the local population was served by 24 general practices. We excluded from the analysis three practices that closed during the follow-up period and a further two practices with incomplete hospital activity data leaving 19 intervention practices. When a practice triggers its closure patients are informed that they should find an alternative local practice to register with. For the three practices that closed we were able to spot patients transferring to one of the other practices retained in the study. These transfers occurred in 2017 and 2019. The two practices excluded because of missing data were relatively small practices. The combined number of patients registered with these two practices was less than five per cent of all patients registered with a NEHF practice. One of the excluded practices was located in an area with a very low level of deprivation (compared with the national average) and the other in an area where the level of deprivation was close to the national average.

Supplementary Table A.1: Variables used to identify similar regions. Rates of hospital activity indicate number of events per 10,000 people in the specified age band registered in the region, calculated across each of the 2 years in the pre-intervention period

Variable	Source	Target cohort	Time period
Percentage of females	Office for National Statistics (ONS)		Mid-2015
Percentage of persons aged 0 to 4 years	ONS		Mid-2015
Percentage of persons aged 5 to 14 years	ONS		Mid-2015
Percentage of persons aged 15 to 44 years	ONS		Mid-2015
Percentage of persons aged 45 to 64 years	ONS		Mid-2015
Percentage of persons aged 65 to 74 years	ONS		Mid-2015
Percentage of persons aged 75 to 89 years	ONS		Mid-2015
Rural/urban indicator	2011 Census		2011
Rate of care home beds available according to Care Quality Commission (per 10,000 registered population)	Care Quality Commission (CQC)	Whole population	July 2015
Rate of GPs (FTE) (per 10,000 registered population)	NHS Digital	Whole population	2014–15
Population density (number of people per square kilometre)	ONS		Mid-2015
Percentage of ethnicity recorded as White	2011 Census		2011
Percentage of ethnicity recorded as Black	2011 Census		2011
Percentage of ethnicity recorded as Asian	2011 Census		2011
Percentage of ethnicity recorded as Mixed	2011 Census		2011
Percentage of ethnicity recorded as Other	2011 Census		2011
Individuals' day-to-day activities limited a lot or a little (standardised illness ratio)	2011 Census		2011
Index of multiple deprivation (IMD) quintile	Index of Multiple Deprivation (IMD)		2015
Health deprivation and disability score	IMD		2015
Income Deprivation Affecting Older People Index (IDAOPI) score	IMD		2015
Rate of yearly emergency admissions for chronic ambulatory care sensitive (ACS) conditions	Secondary Uses Service	age 65+	2013-08-01 to 2015-07-31

	(SUS)		
Rate of yearly emergency admissions for acute ACS conditions	SUS	age 65+	As above
Rate of yearly elective admissions	SUS	age 65+	As above
Rate of yearly emergency admissions	SUS	age 65+	As above
Rate of yearly outpatient appointments attended	SUS	age 65+	As above
Rate of yearly ED visits	SUS	age 65+	As above
Rate of yearly emergency admissions for chronic ACS conditions	SUS	age 18-64	As above
Rate of yearly emergency admissions for acute ACS conditions	SUS	age 18-64	As above
Rate of yearly elective admissions	SUS	age 18-64	As above
Rate of yearly emergency admissions	SUS	age 18-64	As above
Rate of yearly outpatient appointments attended	SUS	age 18-64	As above
Rate of yearly ED visits	SUS	age 18-64	As above
Rate of yearly emergency admissions for chronic ACS conditions	SUS	age 0-17	As above
Rate of yearly emergency admissions for acute ACS conditions	SUS	age 0-17	As above
Rate of yearly elective admissions	SUS	age 0-17	As above
Rate of yearly emergency admissions	SUS	age 0-17	As above
Rate of yearly outpatient appointments attended	SUS	age 0-17	As above
Rate of yearly ED visits	SUS	age 0-17	As above

Supplementary Table A.2: Variables used to identify similar GP practices

Variable	Description	Time period	Level	Source
Population size	Number of registered patients	By quarter, 2013-15	GP practice	NHS Digital
Age	Proportion of registered patients of 18–24, 25–64, 65–74 years of age and of 75 years of age or over	Annual	GP practice	NHS Digital
Gender	Proportion of registered male patients	Annual	GP practice	NHS Digital
Ethnicity	Proportion of registered patients with self-reported White, Black, Asian and Mixed ethnicity	2011 Census	GP practice	Office for National Statistics (ONS)
Education	Proportion of registered patients with at least third-level education (i.e., two or more A levels or equivalent)	2011 Census	GP practice	ONS
Population density	Rate of persons per hectare in the nearest electoral ward	2011 Census	GP practice	ONS
Socio-economic deprivation	Weighted average of lower super output area (LSOA) Index of Multiple Deprivation (IMD) scores according to the LSOA of GP registered patients	2015	Lower super output area (LSOA)	Ministry of Housing, Communities and Local Government
Health deprivation	Weighted average of LSOA IMD scores on health deprivation according to LSOA of GP registered patients	2015	LSOA	Ministry of Housing, Communities and Local Government
Number of full-time equivalent general practitioners	Rate of full-time equivalent general practitioners per 10,000 people in GP population	2015	GP practice	NHS Digital
Quality and Outcomes Framework (QOF) achievement score proportions	The proportion of points achieved, out of total number of points possible, for each Quality and Outcomes Framework (QOF) indicator. These include cardiovascular, respiratory, high dependency and other long-term conditions, and musculoskeletal QOF indicator groups	Annual, 2014-15	GP practice	NHS Digital

QOF disease prevalence	Proportion of registered population with atrial fibrillation, coronary heart disease, cardiovascular disease, heart failure, hypertension, peripheral arterial disease, stroke and transient ischemic attack, asthma, COPD, cancer, chronic kidney disease (18 years of age or over only), diabetes, palliative care, osteoporosis (of 50 years of age or over only) and rheumatoid arthritis (of 16 years of age or over)	Annual, 2014-15	GP practice	NHS Digital
History of Elixhauser comorbidity categories	Total number of emergency admission spells in a given year for patients with a history of each of the Elixhauser comorbidity categories (one variable per comorbidity in the Elixhauser list) per 10,000 of the registered GP population. Comorbidity flags are computed according to primary and secondary diagnosis ICD-10 classification codes for all their inpatient admissions in the preceding 24 months for all patients with emergency admission activity in the month	Annual, 2014-15	Activity	Secondary Uses Service (SUS)
History of Elixhauser Index ≥ 2	Total number of emergency admission spells in a given year for patients with an Elixhauser index ≥ 2 per 10,000 of the registered GP population. Comorbidity flags are computed according to primary and secondary diagnosis ICD-10 classification codes for all their inpatient admissions in the preceding 24 months for all patients with emergency admission activity in the month	Annual, 2014-15	Activity	SUS
History of dementia	Total number of emergency admission spells for patients with dementia per 10,000 of the registered GP population. The dementia flag is computed according to primary and secondary diagnosis ICD-10 classification code in any of their inpatient admissions in the preceding 24 months for all patients with activity in the month	Annual, 2014-15	Activity	SUS
Proportion of inpatient admissions by	Total number of emergency admission spells for patients with positive flags for 22 categories of primary and secondary	Annual, 2014-15	Activity	SUS

primary diagnosis	diagnosis ICD-10 classification code in the preceding 24 months per 10,000 of the registered GP population			
History of emergency admissions	Total number of emergency admission spells in the preceding 24 months per 10,000 people in the registered GP population	Annual, 2014-15	Activity	SUS
History of ED visits	Total number of Ed visits in the preceding 24 months per 10,000 people in the registered GP population	Annual, 2014-15	Activity	SUS

Risk adjustment

Generalised synthetic controls (GSC) allows for the inclusion of time-varying covariates. Two groups of risk adjustment variables were used depending on the outcome. For ED visits and emergency admissions outcomes we adjusted for differences in the characteristics of general practice populations (see Supplementary Table 3). For the average length of stay outcome we adjusted for differences in patients admitted to hospital (see Supplementary Table 4).

Supplementary Table A.3: Variables used for risk adjusting ED visits and emergency admissions

Variable	Description	Time period	Level	Source
Population size	Number of registered patients	By quarter, 2013-20	GP practice	NHS Digital
Age	Proportion of registered patients of 18–24, 25–64, 65–74 years of age and of 75 years of age or over	Annual, 2013-20	GP practice	NHS Digital
Gender	Proportion of registered male patients	Annual, 2013-20	GP practice	NHS Digital
Ethnicity	Proportion of registered patients with self-reported White, Black, Asian and Mixed ethnicity	2011 Census	GP practice	Office for National Statistics (ONS)
Education	Proportion of registered patients with at least third-level education (i.e., two or more A levels or equivalent)	2011 Census	GP practice	Office for National Statistics (ONS)
Population density	Rate of persons per hectare in the nearest electoral ward	2011 Census	GP practice	ONS
Socio-economic deprivation	Weighted average of lower super output area (LSOA) Index of Multiple	2015	Lower super	Ministry of Housing,

	Deprivation (IMD) scores according to the LSOA of GP registered patients		output area (LSOA)	Communities and Local Government
Health deprivation	Weighted average of LSOA IMD scores on health deprivation according to LSOA of GP registered patients	2015	LSOA	Ministry of Housing, Communities and Local Government
Number of full-time equivalent general practitioners	Rate of full-time equivalent general practitioners per 10,000 people in the registered GP population	2015	GP practice	NHS Digital
Quality and Outcomes Framework (QOF) achievement score proportions	The proportion of points achieved, out of total number of points possible, for each Quality and Outcomes Framework (QOF) indicator. These include cardiovascular, respiratory, high dependency and other long-term conditions, and musculoskeletal QOF indicator groups	Annual, 2013-19	GP practice	NHS Digital
QOF disease prevalence	Proportion of registered population with atrial fibrillation, coronary heart disease, cardiovascular disease, heart failure, hypertension, peripheral arterial disease, stroke and transient ischemic attack, asthma, COPD, cancer, chronic kidney disease (18 years of age or over only), diabetes, palliative care, osteoporosis (of 50 years of age or over only) and rheumatoid arthritis (of 16 years of age or over)	Annual, 2013-19	GP practice	NHS Digital

Supplementary Table A.4: Variables used for risk adjusting average length of stay following emergency admission

Variable	Description	Time period	Level	Source
Age	Total number of emergency admission spells for patients of 65-74 years of age, and 75 years of age or over per 10,000 patients admitted to hospital as an emergency	Monthly, 2011-20	Activity	Secondary Uses Service (SUS)
Gender	Total number of emergency admission spells for male patients per 10,000 patients admitted to hospital as an emergency	Monthly, 2011-20	GP Activity	SUS
Ethnicity	Total number of emergency admission spells for patients with self-reported race White, Black, Asian, and Mixed per 10,000 patients admitted to hospital as an emergency	Monthly, 2011-20	Activity	SUS
Proportion of inpatient admissions by primary diagnosis	Total number of emergency admission spells for patients with positive flags for 22 categories of ICD-10 primary and secondary diagnosis classification codes in the preceding 24 months per 10,000 patients admitted to hospital as an emergency	Monthly, 2011-20	Activity	SUS

B. Additional Results

Supplementary Figure B.1: Counterfactual and intervention averages, and average effect, 18+ year-old population (admissions outcomes and ED visits are rates per 10,000 population per month; average length of stay is days). CACSC = chronic ambulatory care sensitive conditions, UCSC = urgent care sensitive conditions.

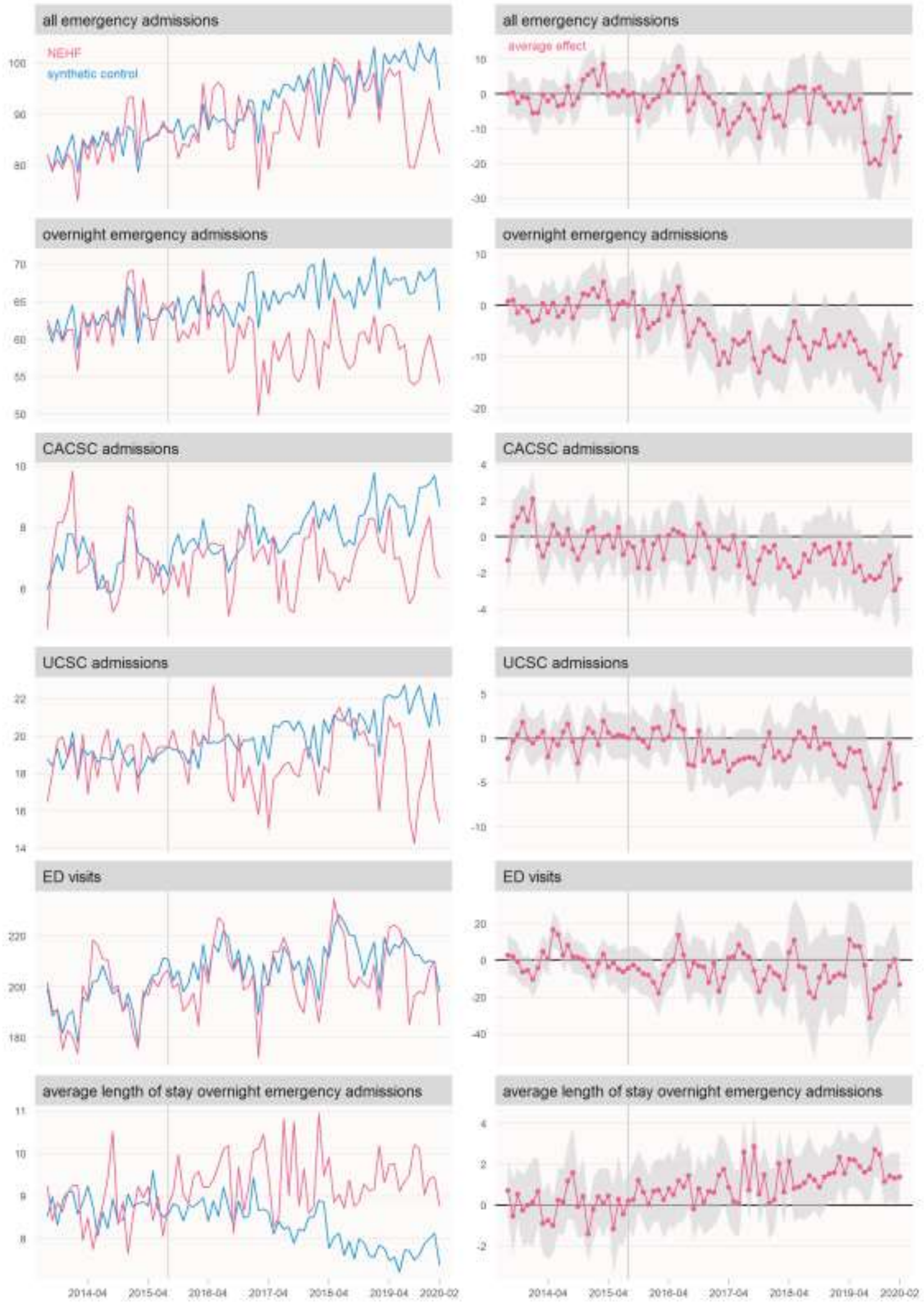
all emergency admissions
NEHF
synthetic control
average effect
overnight emergency admissions
CACSC admissions
UCSC admissions
ED visits
average length of stay overnight emergency admissions
2014-04
2015-04
2016-04
2017-04
2018-04
2019-04
2020-02

Supplementary Table B.1: Average effect (difference between NEHF and estimated counterfactual), 18+ year-old population. Admissions outcomes and ED visits are rates per 10,000 population per month; average length of stay is days. CACSC = chronic ambulatory care sensitive conditions, UCSC = urgent care sensitive conditions.

**Outcome variable**	**Year 1 Aug-15 to Mar-16**	**Year 2 2016-17**	**Year 3 2017-18**	**Year 4 2018-19**	**Year 5 Apr-19 to Feb-20**
All emergency admissions, rate					
Difference	–1.4 (–4.7 to 2.9)	–0.1 (–4.6 to 4.6)	–6.8 (–12.2 to –1.4)	–1.4 (–8.7 to 5.4)	–11.7 (–20.0 to –2.2)
Rel. difference (%)	–1.6 (–5.2 to 3.4)	–0.1 (–4.9 to 5.5)	–7.2 (–12.1 to –1.6)	–1.4 (–8.4 to 5.9)	–11.7 (–18.4 to –2.4)
Overnight emergency admissions, rate					
Difference	–1.6 (–4.7 to 1.4)	–4.3 (–7.9 to –0.7)	–9.2 (–12.8 to –5.5)	–7.0 (–11.6 to –3.0)	–9.8 (–15.0 to –5.0)
Rel. difference (%)	–2.6 (–6.9 to 2.3)	–6.6 (–11.6 to –1.1)	–13.8 (–18.2 to –8.8)	–10.5 (–16.2 to –4.8)	–14.5 (–20.6 to –7.9)
CACSC admissions, rate					
Difference	–0.8 (–1.5 to –0.1)	–0.3 (–1.1 to 0.5)	–1.1 (–1.9 to –0.1)	–1.2 (–2.2 to 0.0)	–1.9 (–3.1 to –0.6)
Rel. difference (%)	–10.6 (–18.9 to –1.3)	–4.5 (–13.7 to 8.1)	–14.4 (–22.0 to –1.5)	–14.3 (–24.1 to –0.4)	–21.6 (–30.8 to –7.5)
UCSC admissions, rate					
Difference	0.2 (–1.0 to 1.8)	–0.9 (–2.5 to 0.8)	–2.1 (–4.2 to –0.2)	–1.0 (–3.5 to 1.4)	–3.8 (–6.4 to –0.7)
Rel. difference (%)	1.0 (–5.1 to 10.0)	–4.5 (–11.9 to 4.7)	–10.5 (–19.0 to –1.0)	–4.7 (–15.0 to 7.7)	–17.4 (–26.1 to –3.9)
ED visits, rate					
Difference	–8.2 (–15.4 to 2.5)	–3.6 (–14.6 to 9.1)	–4.4 (–17.2 to 6.9)	–6.7 (–26.5 to 14.1)	–6.1 (–23.3 to 12.1)
Rel. difference (%)	–4.0 (–7.2 to 1.3)	–1.7 (–6.6 to 4.6)	–2.1 (–7.8 to 3.5)	–3.1 (–11.2 to 7.2)	–2.9 (–10.2 to 6.3)
Average length of stay overnight emergency admissions, days					
Difference	0.5 (–0.2 to 1.2)	0.8 (0.2 to 1.7)	1.0 (0.3 to 1.9)	1.4 (0.7 to 2.2)	1.8 (1.2 to 2.6)
Rel. difference (%)	5.7 (–2.2 to 15.1)	9.6 (2.6 to 21.3)	12.2 (3.4 to 25.7)	18.0 (7.8 to 31.4)	23.9 (14.5 to 36.8)

Supplementary Figure B.2: Average effect (difference between NEHF and estimated counterfactual), 18+ year-old population. Admissions outcomes and ED visits are rates per 10,000 population per month; average length of stay is days. Red = confidence interval does not contain zero, blue = confidence interval contains zero. CACSC = chronic ambulatory care sensitive conditions, UCSC = urgent care sensitive conditions.

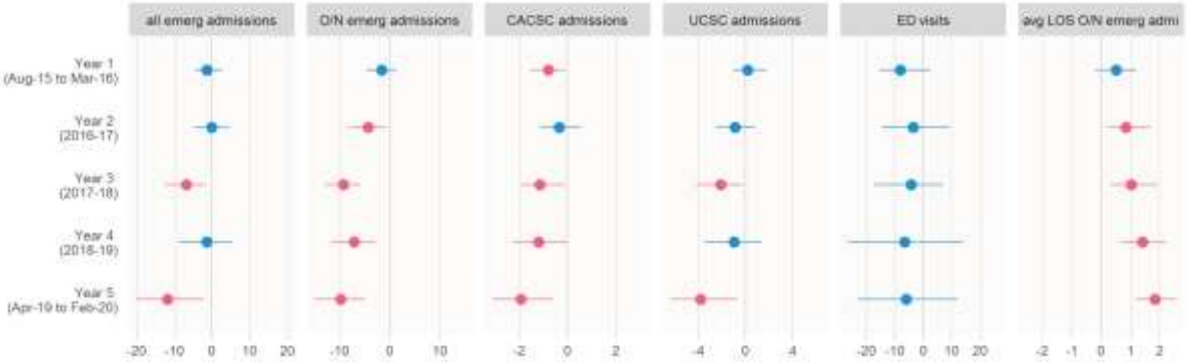

Supplement: Appendix. — Supplementary materials. [file ijic-23-3-6475-s1.pdf]
